# Inflammatory Fibroid Polyp: An Unusual Cause of Ileoileal Intussusception

**DOI:** 10.1155/2017/6315934

**Published:** 2017-11-16

**Authors:** Haley S. Adams, Brian Bergstrom, Bret Haines, Nathan Roberts

**Affiliations:** Department of Surgery, Oklahoma State University Medical Center, Tulsa, OK, USA

## Abstract

Inflammatory fibroid polyp (IFP), or Vanek's tumor, is a rare benign lesion of the gastrointestinal tract. Clinical manifestations of IFP vary based on size and location within the GI tract. This case describes a patient who presented with hematochezia and abdominal pain. Computed tomography revealed ileoileal intussusception without a clear lead point. The patient underwent resection of the intussuscepted small bowel with primary anastomosis. A large polypoid mass was identified as the pathological lead point. Histopathological and immunohistochemical analysis revealed an IFP. Review of the literature indicates that early surgical intervention is the treatment of choice for intussusception caused by IFP. Lesions are typically reported as solitary, and resection is curative.

## 1. Introduction

Vanek's tumor, or inflammatory fibroid polyp (IFP), is one of the least common benign small bowel tumors [1, 2]. This rare lesion can be found throughout the gastrointestinal (GI) tract but is most commonly found in the gastric antrum [3]. Peak incidence occurs in the sixth and seventh decades of life, with a slight male predominance [4]. Clinical manifestations of IFP depend largely on size and location, but most commonly include intestinal obstruction, abdominal pain, intussusception, and rarely GI bleeding [5, 6]. We herein present a patient with hematochezia, intermittent abdominal pain, and ileoileal intussusception. Given intraoperative concern for a malignant lead point, small bowel resection with wide margins was performed. Final pathology yielded a diagnosis of Vanek's tumor.

## 2. Case Report

**Figure 1:**
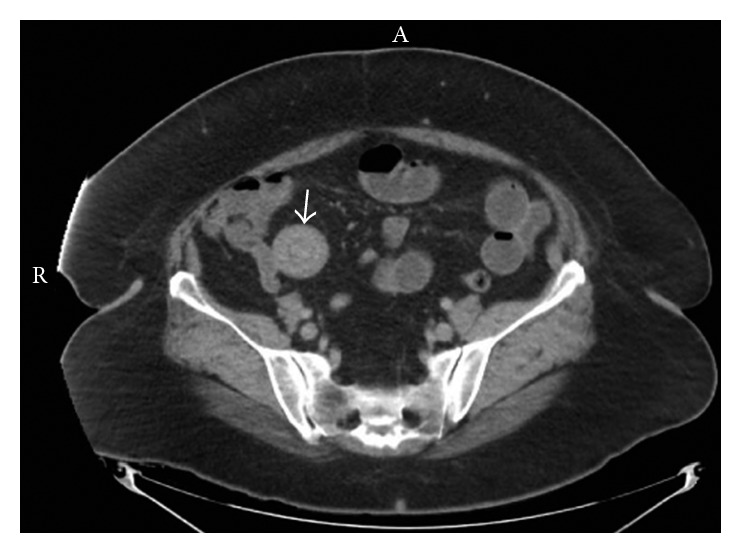
Axial abdominal computed tomography revealing ileoileal intussusception (arrow).

**Figure 2:**
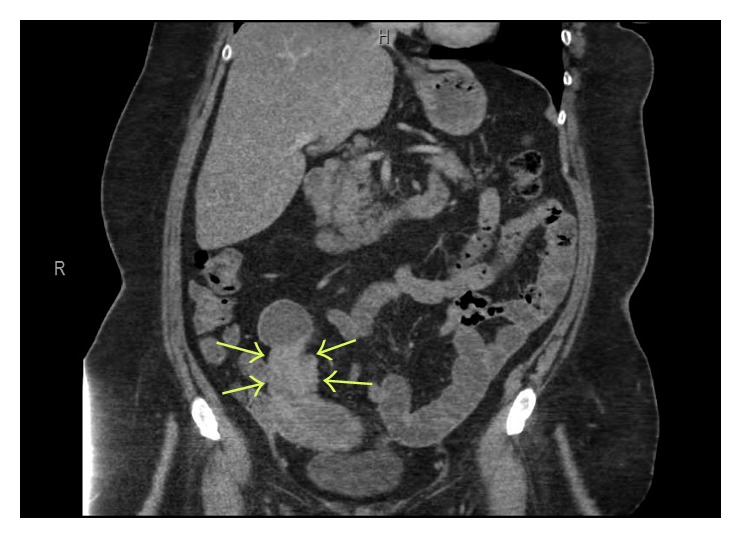
Coronal abdominal computed tomography revealing ileoileal intussusception (arrows).

A 61-year-old female presented to our facility with a two-week history of diarrhea and hematochezia, and a two-day history of intermittent severe right lower quadrant pain. The patient had a significant recent history of percutaneous coronary intervention with stent placement and was on dual antiplatelet therapy with aspirin and ticagrelor. Upon physical exam, her vital signs were stable and she was in no acute distress. Abdominal examination revealed mild distension, hyperactive bowel sounds, and significant tenderness upon palpation of the right lower quadrant. The patient did not exhibit guarding or rebound tenderness. Laboratory investigation showed leukocyte count 12.9 × 10^9^ cells/L (normal: 4–12 × 10^9^ cells/L), hemoglobin 9.7 g/dL (normal: 11.1–15.5 g/dL), and platelet count 555 × 10^3^ cells/L (normal: 130–400 × 10^3^ cells/L). Serum chemistry values, including lactic acid, were within normal limits with the exception of glucose 172 mg/dL (normal: 71–110 mg/dL). Computed tomography of the abdomen and pelvis with intravenous contrast revealed ileoileal intussusception with local inflammatory changes and proximal early small bowel obstruction (Figures 1 and 2). No nidus or lead point for the intussusception could be clearly identified on imaging.

**Figure 3:**
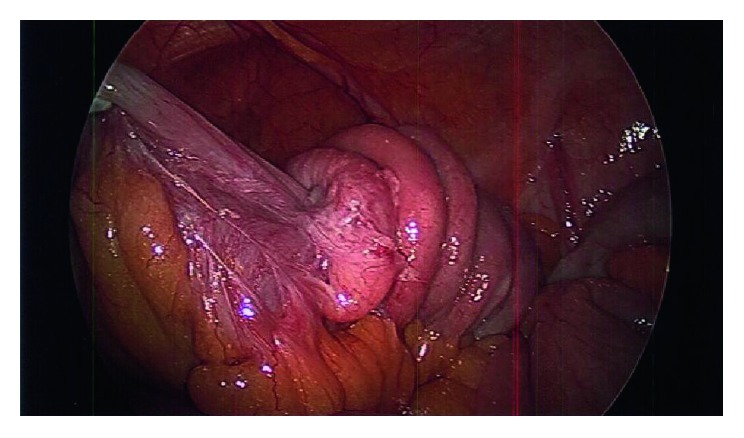
Intraoperative laparoscopic view of the intussuscepted portion of the ileum, showing dense fixation at the lead point.

The patient was taken to the operating room for a diagnostic laparoscopy. Intraoperatively, a segment of decompressed small bowel approximately 30 cm proximal to the ileocecal valve was noted to be tethered in the pelvis. Adjacent to this adhesion, the intussuscepted segment of the ileum was identified (Figure 3). Limited manipulation of this segment revealed concern for a mass within the small intestine. An infraumbilical minilaparotomy was made, and the small bowel was extracorporealized. Upon further examination, the adjacent mesentery was noted to have several palpable lymph nodes. A segmental resection of the involved ileum with associated mesentery was performed, and a side-to-side stapled anastomosis was created in the standard fashion.

**Figure 4:**
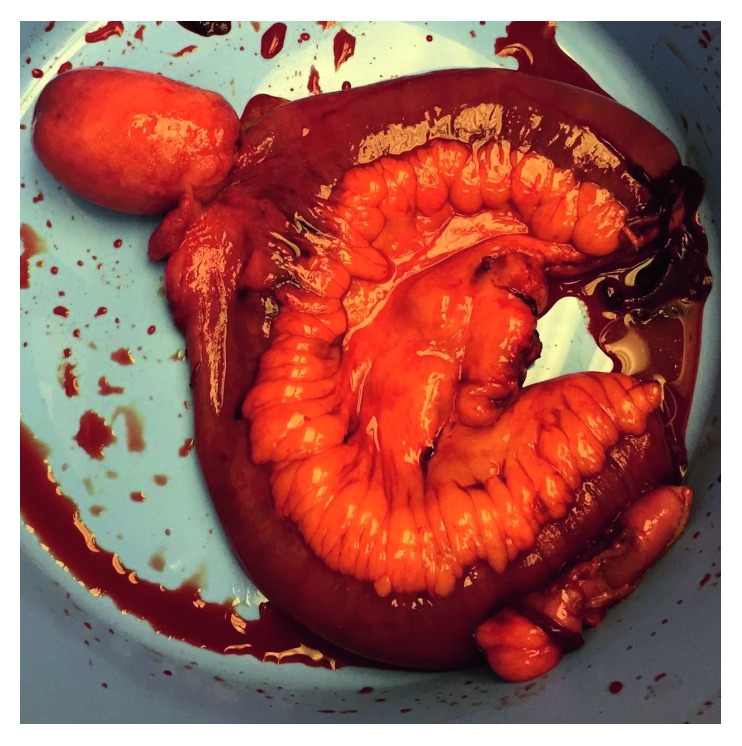
Postoperative specimen. After segmental small bowel resection was performed, the intussuscepted segment was reduced on the back table. The polyp measured 7.5 × 4.5 × 3.2 cm grossly.

Once removed, the ileal specimen was examined on the back table. The intussusception was reduced, at which time a large polypoid lesion was identified (Figure 4). The mass appeared grossly to be extraluminal. The abdominal wall was closed in layers, and the patient was sent to the general medical floor postoperatively. The patient tolerated the procedure well, had an uneventful immediate postoperative course, and was discharged on the fourth postoperative day.

**Figure 5:**
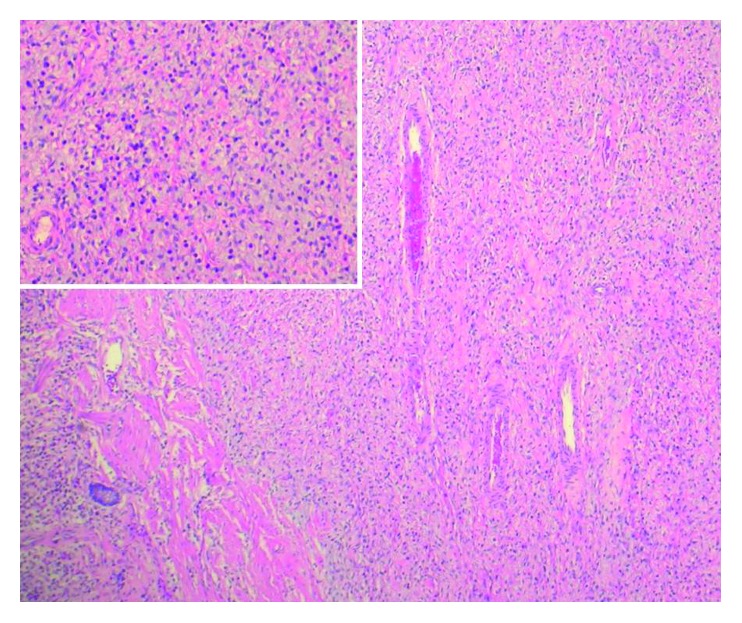
Photomicrograph of small bowel polyp. H&E staining demonstrates myxedematous stroma with prominent vessels and a striking inflammatory infiltrate composed of abundant eosinophils. Inset shows magnification.

**Figure 6:**
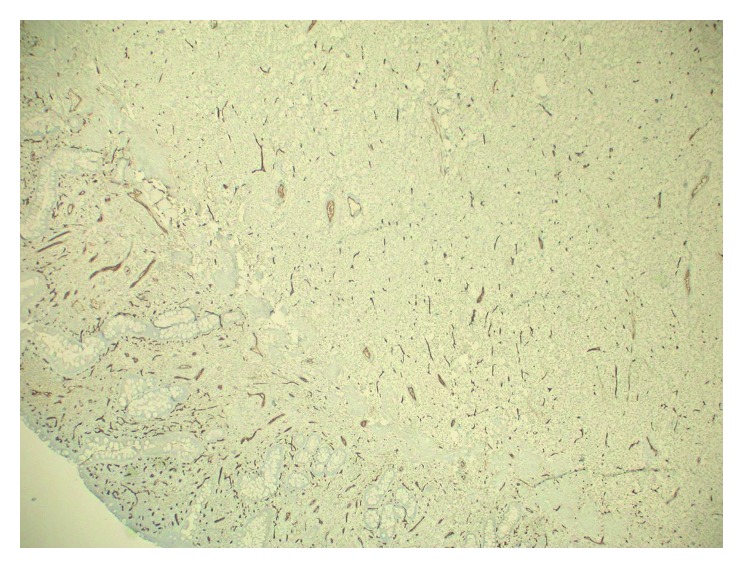
Photomicrograph of surgical specimen demonstrating negative immunostaining with CD34.

Grossly, the resected segment of the ileum was 22 cm in length with a firm polypoid lesion measuring 7.5 × 4.5 × 3.2 cm identified at 9.5 cm from the proximal margin. The subjacent mucosa was noted to be edematous and hyperemic. Upon microscopic examination, the specimen was noted to be a well-circumscribed submucosal-based lesion having marked myxedematous stroma with prominent vessels and a background of spindle cells without atypia or necrosis. There was a striking inflammatory infiltrate composed of abundant eosinophils as well as frequent plasma cells and occasional neutrophils (Figure 5). The overlying mucosal surface was ulcerated with foci of erosion and chronic active inflammation. No evidence of overlying dysplasia or malignancy was found. Six adjacent lymph nodes were identified and microscopically demonstrated reactive follicular hyperplasia without evidence of neoplasia. On immunohistochemical analysis, spindle cells were negative for CD117, smooth muscle actin, desmin, S100 protein, DOG1, and CD34 (Figure 6). Cellular proliferation was studied using Ki-67, which showed rare mitotic figures. The morphological features were typical of IFP, and the immunoprofile is consistent with that diagnosis.

Of note, the patient did present with a superficial surgical site infection one week postoperatively, which was successfully treated with oral antibiotics and local wound care. The patient's BMI (40.5 kg/m^2^) and poorly controlled insulin-dependent diabetes mellitus type II (HgbA1c 9%) were likely significant contributors to this complication. Additionally, she presented with one episode of painless hematochezia two weeks postoperatively. Esophagogastroduodenoscopy and colonoscopy were performed but failed to show evidence of active GI bleeding. At four-month follow-up, the patient was well healed without recurrence of symptoms.

## 3. Discussion

IFPs are rare clinically benign mesenchymal tumors originating in the submucosa of the gastrointestinal tract. The incidence of IFP is unknown. First described as “gastric submucosal granuloma(s) with eosinophilic infiltration” by Vanek in 1949, these lesions have subsequently been identified throughout the GI tract [3]. The most common site is the gastric antrum (60–70%), followed by small bowel (18–20%), colorectum (4–7%), and far less commonly (1%) in esophagus, duodenum, gallbladder, and appendix [2]. The polyps are typically solitary, but rare metachronous lesions have been reported in familial cases [7, 8]. Most IFPs grow intraluminally and are smaller than 4 cm, but case reports have discussed polyps up to 20 cm [9]. In this case, the polyp measured 7.5 cm in greatest dimension, with the majority of the mass extending extraluminally.

Clinical manifestations depend largely on tumor location and size. Often IFPs are asymptomatic and are identified incidentally during endoscopic or surgical procedures. Abdominal pain and nausea are the most frequent symptoms in patients with gastric lesions [2]. In contrast, patients with IFP in the small bowel are more likely to present with chronic colicky abdominal pain, small bowel obstruction, intussusception, and weight loss [1]. GI bleeding is a rare presenting symptom, and if present, it may indicate significant ulceration or ischemia [7].

Our patient presented with a two-week history of bleeding, which appears to be unique amongst published reports. The microscopic examination of our resected specimen did show that the overlying luminal surface was ulcerated with foci of erosion, which in combination with the patient's dual antiplatelet therapy likely resulted in prolonged duration of gastrointestinal bleeding.

Historically, IFPs were thought to represent a reactive inflammatory process with trauma, allergic reaction, and bacterial, physical, chemical, or metabolic stimuli, all suggested as inciting events [1]. More recently, reports of familial occurrence and recognition of activating platelet-derived growth factor receptor alpha (PDGFRA) mutations in these tumors suggest that IFPs represent true neoplasms [8]. Similar mutations in the PDGFRA gene have been found in gastrointestinal stromal tumors (GISTs), which imply a common oncogenetic pathway [10]. Immunohistochemically, IFPs are negative for CD117 and variably positive for CD34 [11]. Negative CD34 immunoreactivity, as was seen in our case, is demonstrated in 10–15% of IFPs [12, 13]. In contrast, GISTs have characteristically positive CD117 and CD34 immunostaining [11]. IFPs are benign neoplasms and are generally considered to have no risk of recurrence or metastasis after removal.

In adults, a lead point is responsible for at least 65% of all intussusceptions [6]. A variety of benign and malignant lesions can cause intussusception [1]. Benign lead points may be related to intra-abdominal adhesions or masses such as lipomas, leiomyomas, neurofibromas, adenomas, and more rarely IFP [6].

The clinical history of intussusception often mirrors bowel obstruction and includes episodic abdominal cramping, nausea, and vomiting. Compared to pediatric patients, GI bleeding occurs less often in adult patients with intussusception but may suggest bowel wall ischemia if present [4]. Abdominal CT is the preferred imaging technique for adults with intussusception, with sensitivity between 50 and 100%; however, visualization of a mass within the intussusception is rare [14].

Depending on the location, IFP may be diagnosed and treated endoscopically. Several reports of endoscopic resection of IFPs in the stomach, duodenum, and colon have been published [15, 16]. Historically, surgical resection is the treatment of choice for symptomatic IFPs. Resection is curative, and only one case of polyp recurrence is found in the literature [2].

In the adult population, once intussusception is diagnosed, prompt surgical intervention is warranted to avoid complications of ischemia, necrosis, and perforation. Debate in the literature on appropriate surgical treatment for adult intussusception focuses largely on initial resection of the intussuscepted segment versus reduction followed by a more limited resection. Current recommendations favor reduction and limited resection in small bowel intussusception, but only if the bowel is easily reduced and the lead point appears grossly benign [17]. In our patient, wide resection of the intussuscepted segment was performed, as there was significant intraoperative concern for malignancy, given the gross appearance of the segment and the associated mesenteric lymphadenopathy.
